# The long-term mental health impact of peacekeeping: prevalence and predictors of psychiatric disorder

**DOI:** 10.1192/bjpo.bp.115.001321

**Published:** 2016-01-20

**Authors:** David Forbes, Meaghan O’Donnell, Rachel M. Brand, Sam Korn, Mark Creamer, Alexander C. McFarlane, Malcolm R. Sim, Andrew B. Forbes, Graeme Hawthorne

**Affiliations:** David Forbes, PhD; **Meaghan O’Donnell**, PhD; **Rachel M. Brand**, DClinPsy, Phoenix Australia – Centre for Posttraumatic Mental Health, Department of Psychiatry, University of Melbourne, Melbourne, Victoria, Australia; **Sam Korn**, PhD, Mental Health Evaluation Unit, Department of Psychiatry, University of Melbourne, Melbourne, Victoria, Australia; **Mark Creamer**, PhD, Phoenix Australia – Centre for Posttraumatic Mental Health, Department of Psychiatry, University of Melbourne, Melbourne, Victoria, Australia; **Alexander C. McFarlane**, AO, MD, FRANZCP, Centre for Traumatic Stress Studies, University of Adelaide, South Australia, Australia; **Malcolm R. Sim**, PhD; **Andrew B. Forbes**, PhD, Monash Centre for Occupational & Environmental Health, Department of Epidemiology & Preventive Medicine, Faculty of Medicine, Nursing & Health Sciences, Monash University, Melbourne, Victoria, Australia; **Graeme Hawthorne** (deceased), PhD, Mental Health Evaluation Unit, Department of Psychiatry, University of Melbourne, Melbourne, Victoria, Australia

## Abstract

**Background:**

The mental health outcomes of military personnel deployed on peacekeeping missions have been relatively neglected in the military mental health literature.

**Aims:**

To assess the mental health impacts of peacekeeping deployments.

**Method:**

In total, 1025 Australian peacekeepers were assessed for current and lifetime psychiatric diagnoses, service history and exposure to potentially traumatic events (PTEs). A matched Australian community sample was used as a comparator. Univariate and regression analyses were conducted to explore predictors of psychiatric diagnosis.

**Results:**

Peacekeepers had significantly higher 12-month prevalence of post-traumatic stress disorder (16.8%), major depressive episode (7%), generalised anxiety disorder (4.7%), alcohol misuse (12%), alcohol dependence (11.3%) and suicidal ideation (10.7%) when compared with the civilian comparator. The presence of these psychiatric disorders was most strongly and consistently associated with exposure to PTEs.

**Conclusions:**

Veteran peacekeepers had significant levels of psychiatric morbidity. Their needs, alongside those of combat veterans, should be recognised within military mental health initiatives.

**Declaration of interest:**

None.

**Copyright and usage:**

This is an open access article distributed under the terms of the Creative Commons Attribution (CC BY) licence.

The mental health outcomes of military personnel following combat have been widely documented in a large body of high-quality, international research.^1–7^ This body of research is bolstered by a small but growing number of methodologically rigorous studies examining the prevalence of psychiatric disorder across currently serving military populations.^8,9^ In contrast, however, research dedicated to the mental health outcomes of military personnel deployed on peacekeeping missions has been limited.

United Nations (UN)-sanctioned peacekeeping missions can vary significantly in complexity, demands and potential hazards. Many peacekeeping missions may involve experiences not unlike combat deployments. Military personnel deployed on peacekeeping missions may encounter numerous stressful and potentially traumatic events (PTEs), such as delivering humanitarian aid amidst volatile environments, coming under hostile fire, witnessing atrocities against civilians and handling dead bodies.^10–12^ In addition, peacekeepers may face the challenge of operating under restrictive rules of engagement, which can lead to role conflict and ambiguity regarding appropriate action under situations of threat to themselves or others.^13^

A systematic review examining the association between deployment on a peacekeeping mission and subsequent psychiatric morbidity and distress found that the small body of research published in this area has been equivocal.^14^ Similarly, a meta-analysis of 12 studies examining rates of post-traumatic stress disorder (PTSD) more specifically in peacekeepers found a pooled point PTSD prevalence of 5.3%, with study prevalence rates being very variable, ranging from 1% to 25.8%.^15^ The field has been highlighted as lacking methodologically rigorous studies, with all bar one^9^ study relying on self-report measures only. Given the nature of peacekeeping experiences and the growing international participation in such deployments, it is critical that methodologically rigorous research be conducted to examine their long-term mental health effects. This study is the first comprehensive, methodologically rigorous study of the long-term mental health outcomes of veteran peacekeepers using robust assessment methods. The use of a matched civilian comparator group also provides a unique addition to the evidence base regarding peacekeeper mental health outcomes.

## Method

### Participants

Participants were Australian peacekeepers who had deployed on one or more of seven UN-sanctioned interventions between 1989 and early 2002. The selected deployments were those to which Australia had contributed significant contingents of peacekeepers: Namibia (1989–1990, United Nations Transition Assistance Group; *n*=613), Western Sahara (1991–1994, Mission for the Referendum in Western Sahara; *n*=225), Cambodia (1991–1993, United Nations Advance Mission in Cambodia/United Nations Transitional Authority in Cambodia; *n*=1215), Rwanda (1994–1995, United Nations Assisstance Mission for Rwanda II; *n*=638), Somalia (1992–1996, United Nations Operation in Somalia I/United Task Force Somalia/United Nations Operation in Somalia II; *n*=1480), East Timor (1999, International Forces East Timor; *n*=7970) and East Timor (1999–2002, United Nations Transitional Administration in East Timor; *n*=2090). These deployments encompass both peacekeeping and peace-enforcing missions. Many of these missions involved operations in very challenging and dangerous environments.

Of those who were eligible (identified by the Department of Veterans’ Affairs (DVA) and Australian Defence Force (ADF) from their records), random stratified sampling was used to select 2247 peacekeepers to be invited to participate. This sample size was ascertained using power analyses, plus oversampling by 50% to allow for low participation rates. This base sample was then increased by 25% to avoid reduced statistical power due to clustering.

### Comparator sample

To allow for comparisons with the civilian population, a matched sample was drawn from the 2007 National Survey of Mental Health and Wellbeing (NSMHWB).^16^ This was a large-scale epidemiological study which investigated the prevalence of common psychiatric disorders in the Australian general population. The full NSMHWB sample was a nationally representative sample of 8841 people aged 16–85 living in Australia, selected through random, stratified, multistage area sampling. Confidentiality restrictions prohibited any attempt to match NSMHWB data at an individual level; therefore, matching was carried out at an aggregate level. Aggregate variables of interest from the peacekeepers’ data were matched with variables from the NSMHWB where these variables were collapsed to form suitable aggregates. The variables used were gender (male/female), age (5-year cohorts), relationship status (single/partnered/separated or divorced or widowed) and education attainment (primary/high/trade or technical and further education (TAFE)/college or university degree). The NSMHWB database (excluding veterans) was searched for matching logical aggregate groups and group membership adjusted to achieve a 1:1 relationship with study peacekeepers through randomly sampling the necessary proportion to achieve similar sized groups. This resulted in a comparison group matched for gender, age, education and relationship status against which to compare study peacekeeper participants.

### Measures

The prevalence of current (within the past 12 months) and lifetime psychiatric diagnoses in the peacekeeper sample were assessed using the World Mental Health Survey Initiative version of the World Health Organization's Composite International Diagnostic Interview, Version 3.0 (CIDI).^17^ The CIDI 3.0 has excellent psychometric properties.^17^ A modified Australian version of the CIDI was used for the NSMHWB and was therefore also used in the peacekeeper sample. Diagnosis using the CIDI was based on the ICD-10 diagnostic algorithm.^18^ To reduce burden on participants, four diagnostic modules were administered to the peacekeepers: PTSD, major depressive episode, generalised anxiety disorder and alcohol/substance use disorders. These four modules were selected because previous research with Australian veterans has identified these conditions as being likely to show elevated rates. Suicidal ideation, plans and attempts were also assessed using the CIDI. The CIDI elicits the age at first symptom onset, which was used in combination with the age at first deployment to calculate pre-deployment prevalence. In the civilian comparator sample, the comparison prevalence rates for pre-deployment were calculated by specifying a cut-off for age at onset as 26 years, which was the median age of peacekeepers’ first deployment.

Standardised self-report questionnaires were also administered to the peacekeeper sample to gather demographic information and military service history. Exposures to PTEs generally across the lifespan were measured using the Life Events Checklist (LEC),^19^ a widely used self-report measure designed to screen for PTEs in a respondent's lifetime. The LEC probes experience of 16 PTEs, based on DSM-IV criterion A events. The LEC has been found to have good psychometric properties.^19^ Exposures to PTEs specifically on deployment were measured using the Traumatic Stress Exposure Scale-Revision 2 (TSES-R2).^20^ The TSES-R2 is a 12-item checklist designed to measure the frequency of PTEs during deployment and the severity of associated fear and/or horror experienced at the time of the event and currently. The TSES-R2 was developed with peacekeepers in East Timor and has since been used extensively in the ADF in peacekeeping and combat settings. It has good test–retest reliability.^20^

### Ethics

Ethical approval for the study was obtained from the Australian DVA Human Research Ethics Committee, the ADF Human Research Ethics Committee and The University of Melbourne Human Research Ethics Committee. After a description of the study was mailed to the participants, written informed consent was obtained from all participants by return mail.

### Statistical analysis

Descriptive statistics were used to provide information on the nature and prevalence of key variables. Key comparisons were explored using tests of significance including *t*-tests, analysis of variance, chi-squared and odds ratios. Univariate analyses were used to explore key predictors of psychiatric disorder, with significant variables then entered into a multivariate logistical regression model for the most prevalent disorder, PTSD.

## Results

### Participants

A total of 2247 invitations were mailed to peacekeepers. A total of 163 were returned because the recipient was not known at the address, leaving 2084 letters delivered. Six hundred peacekeepers did not respond to the invitation. The initial participation comprised 1311 peacekeeper veterans who consented to take part (88% of the remaining 1484 who were contactable). The final sample comprised 1025 participants who completed the CIDI (69% of those who were contactable). Information regarding participant demographics is included in Table 1.

**Table 1:** Participant demographics (*n*=1025)

	Total, %
Age group, years	
0–39	19.5
40–49	48.8
50–59	23.8
60+	7.8
Age, years: mean	46.5

Gender	
Male	95.5
Female	4.5

Educational attainment	
Primary school	1.3
Trade certificate	42.0
High school	35.6
University/College degree	21.1

Relationship	
Single	7.0
Married/de facto	81.3
Divorced/separated	11.7

Work status (current)	
Working full or part-time	74.9
Out of workforce	4.9
Retired or on benefits	20.2

Household income, $	
0–24 999	4.0
25 000–49 999	12.2
50 000–74 999	24.7
75 000–99 999	20.4
100 000+	38.5

Pension status	
Receiving benefits	43.4
Not receiving benefits	56.6

Number of times deployed^a^	
1	73.6
2	19.1
3+	7.3

Number of study deployments^b^	
1	87.0
2+	13.0

a Number of times deployed refers to the number of deployments in the participant’s career, including deployments outside of the study deployments.

b Number of study deployments refers solely to the number of times participants have been deployed specifically to the peacekeeping deployments in the scope of this study.

Study participants (i.e. full completers, *n*=1025) were significantly older than non-responders (including those who were not contactable and who failed to complete all measures, *n*=1222, *P*<0.01) and were from a higher socioeconomic background (*P*<0.03). The Somalia and East Timor deployments had significantly lower response rates, whereas higher response rates were obtained from the deployments to Cambodia, Namibia and Western Sahara (*P*<0.01).

### Trauma exposure on deployment

TSES-R2 data were available for 1014 of the participants. These participants reported high levels of exposure to PTEs on deployment, with the most common being threat of injury (83%) or death (77%), seeing dead bodies (78%), witnessing degradation and misery (72%), and hearing of a friend or co-worker being injured or killed (64%). Causing the death (17%) or injury (20%) of another person was the least reported. Most peacekeepers reported being exposed either to particular events multiple times or to multiple types of PTEs.

Details of cumulative exposure to PTEs, based on the scores of the TSES-R2, are provided in Table 2. Notably, there were significant differences in exposure to PTEs by deployment, with those deployed to Somalia and Rwanda reporting the highest numbers. The same pattern of highest reporting in Somalia and Rwanda was observed for the measures of fear and/or horror associated with the exposures at the time and currently.

### Traumatic life events exposure

In terms of overall life experiences, the most common PTEs were transport accidents (56%), physical assaults (49%) and the sudden unexpected death of someone close (41%).

### Criterion A events

Peacekeepers who met criteria for a PTSD diagnosis were significantly more likely than their civilian counterparts to report peacekeeping, combat and witnessing atrocities as the Criterion A event (χ^2^+82.44, d.f.=5, *P*<0.01), with 27% of peacekeepers with PTSD reporting peacekeeping as their index traumatic event, 16% reporting a combat experience and 12% reporting witnessing atrocities.

### Pre-deployment mental health

The prevalence of mental health disorders pre-deployment was very low – significantly lower for PTSD (2.7%), major depressive episode (1.3%) and generalised anxiety disorder (1.1%) than the NSMHWB civilian cohort (6.1%, 5.6% and 3.9% respectively). Pre-deployment alcohol use disorders were significantly more prevalent among peacekeepers (alcohol misuse 34.6%, alcohol dependence 8.7%) than in the civilian sample (29.5% and 3.5% respectively).

### The prevalence of psychiatric disorder

A total of 29.9% of peacekeepers met criteria for at least one CIDI-diagnosed ICD-10 psychiatric disorder. This compares with 12.2% of the civilian NSMHWB sample. As demonstrated in Table 3, psychiatric comorbidity was common in the peacekeeper sample, with 21.6% having one diagnosis, 6.8% having two and 1.5% having three or more. Peacekeepers were significantly more likely to have more disorders than their civilian counterparts.

**Table 2:** Deployment-related traumatic stress exposure assessed by the Traumatic Stress Exposure Scale – R2 (TSES-R2), by deployment^a^

Deployment	*n*	TSES-R2: frequency		TSES-R2: fear and/or horror at the time		TSES-R2: fear and/or horror now	
		Mean	s.d.	Mean	s.d.	Mean	s.d.
Cambodia	293	9.43	4.83	9.00	6.38	5.56	6.54
Namibia	193	8.03	4.91	7.81	5.67	4.70	5.54
Rwanda	110	11.51	5.09	10.92	6.78	8.54	8.09
W. Sahara	38	6.55	5.16	7.61	7.35	5.21	8.11
Somalia	214	11.44	4.81	10.84	6.85	8.21	8.29
INTERFET	65	8.14	5.49	7.58	6.04	3.68	4.87
UNTAET	101	7.37	4.81	6.09	5.09	4.43	5.44
Total	1014	9.42	5.16	8.97	6.48	6.07	7.03
Statistics		Kruskal–Wallis χ^2^=95.28, d.f.=6, *P*<0.01		Kruskal–Wallis χ^2^=59.89, d.f.=6, *P*<0.01		Kruskal–Wallis χ^2^=47.17, d.f.=6, *P*<0.01	

INTERFET, International Forces East Timor; UNTAET, United Nations Transitional Administration in East Timor.

a TSES-R2 data were available for 1014 of the 1025 participants.

**Table 3:** Twelve-month CIDI diagnosed psychiatric disorder comparisons

	PK total sample (*n*=1025), %	NSMHWB sample (*n*=1025), %	Statistics
Post-traumatic stress disorder	16.8	6.0	*χ*^2^=59.66, d.f.=1, *P*<0.01

Generalised anxiety disorder	4.7	2.9	*χ* ^2^=4.32, d.f.=1, *P*=0.04

Major depressive episode	7.0	2.8	*χ* ^2^=19.26, d.f.=1, *P*<0.01

Alcohol misuse	12.0	3.5	*χ* ^2^=51.61, d.f.=1, *P*<0.01

Alcohol dependence	11.3	3.6	*χ* ^2^=44.08, d.f.=1, *P*<0.01

Substance dependence	2.6	0.7	*χ* ^2^=0.78, d.f.=1, *P*=0.38

Suicidal ideation	10.7	2.7	*χ* ^2^=51.35, d.f.=1, *P*<0.01

Suicide plan	5.8	0.7	*χ* ^2^=42.33, d.f.=1, *P*<0.01

Suicide attempt	1.0	0.2	*χ*^2^=5.37, d.f.=1, *P*=0.02

Number of CIDI disorders			
1	21.6	9.7	
2	6.8	1.7	
3+	1.5	0.8	*χ* ^2^=167.05, d.f.=2, *P*<0.01

CIDI, Composite International Diagnostic Interview; NSMHWB, National Survey of Mental Health and Wellbeing; PK, peacekeeper.

Comparisons between the prevalence rates of the peacekeeper sample and the NSMHWB civilian comparator sample are shown in Table 3. Rates for the peacekeepers were significantly higher than their civilian counterparts for all disorders, except substance dependence where the rate was higher but did not reach statistical significance.

Notably, there were no significant differences in rates of psychiatric disorder between different peacekeeping deployments. Peacekeepers deployed to Somalia and Rwanda showed the highest rates of PTSD, but this did not reach statistical significance.

### Suicidality

Twelve-month prevalence frequencies for suicidal ideation, suicide plan and suicide attempt are also shown in Table 3. Overall, the level of suicidality in this sample was high, with 10.7% reporting suicidal ideation, 5.8% a suicide plan and 1% a suicide attempt. This is significantly higher than the NSMHWB civilian cohort.

### Correlates and predictors of psychiatric disorder

Univariate analyses yielded several variables which were significantly correlated with psychiatric outcomes. These are represented in Table 4. Demographic variables showed some significant associations. Income level was significantly related to PTSD and major depressive episode (and less so to alcohol use disorders). Employment status (working *v*. being retired or in receipt of benefits) was strongly associated with all disorders.

**Table 4:** Predictors of psychiatric disorder: univariate analyses, by disorder^a^

		Test statistic (d.f.)				
		
	Statistical test	Post-traumatic stress disorder	Generalised anxiety disorder	Major depressive episode	Alcohol misuse	Alcohol dependence
Demographics						
Marital status: unpartnered	Chi-squared	7.37 (2)*	2.94 (2) ns	2.99 (2) ns	5.32 (2) ns	6.03 (2)*
Age: older	Chi-squared	0.86 (3)*	0.73 (3) ns	1.46 (3) ns	2.03 (3) ns	2.71 (3) ns
Employment: retired/sickness benefit	Chi-squared	40.03 (2)***	37.37 (2)***	52.13 (2)***	13.74 (2)**	10.94 (2)**
Income	Chi-squared	19.72 (4)**	6.88 (4) ns	17.97 (4)**	13.08 (4)*	11.58 (4)*

Trauma exposure/deployments						
Age at deployment: older	Kruskal–Wallis *χ* ^2^	7.50 (1)**	0.45 (1) ns	0.20 (1) ns	0.24 (1) ns	0.11 (1) ns
No. of deployments: >1	Chi-squared	5.23 (1)*	0.24 (1) ns	17.81 (1)***	1.44 (1) ns	1.34 (1) ns
Deployment-related PTEs	Unpaired *t*-test	9.35 (1012)***	5.83 (1012)***	5.33 (1012)***	2.99 (1012)**	9.59 (1012)**
Fear/horror at time	Kruskal–Wallis *χ* ^2^	93.69 (1)***	25.12 (1)***	23.62 (1)***	17.59 (1)***	17.50 (1)***
Lifetime PTEs	Kruskal–Wallis *χ* ^2^	58.13 (1)***	29.67 (1)***	10.37 (1)***	11.51 (1)**	6.85 (1)**

ns, not significant; PTE, potentially traumatic event.

a For the purpose of univariate analyses, demographic variables were categorised as shown in Table 1. Trauma exposure/deployment variables were dichotomised (other than deployment related PTEs which was treated as a continuous variable).

* *P*<0.05;

** *P*<0.01;

*** *P*<0.001.

Both lifetime- and deployment-related trauma exposures were significantly associated with all psychiatric disorders. Similar relationships were found for reported fear or horror at the time of the deployment trauma exposure.

Those participants who had deployed more than once were no more likely than those with single deployment histories to meet criteria for alcohol use disorders or generalised anxiety disorder. There was a significant relationship between the number of deployments and major depressive episode – those veterans with multiple deployments more likely to meet criteria for major depressive episode. Age at the time of deployment was related to PTSD only, with those participants who were older at the time of deployment being more likely to have current PTSD (mean age was 28 years for those with PTSD and 26 years for those without).

Predictors of the most prevalent disorder, PTSD, were explored in further depth using multivariate analyses. When all significant predictor variables from the univariate analyses were combined, the logistic regression model showed that 12-month PTSD was a function of PTE exposure (both deployment and lifetime non-deployment exposure), fear or horror experienced at the time of deployment PTEs and being out of the workforce. This model is shown in Table 5.

**Table 5:** Logistic regression model for 12-month CIDI-diagnosed post-traumatic stress disorder^a^

Predictor	Base	Comparator	Odds ratio	95% CI
Life events checklist	0–4 events	5+ events	2.98	1.85–4.82
Number of exposures from the TSES-R2	0–9 events	10+ events	1.60	1.00–2.54
Fear and/or horror from deployment exposures at the time	No feelings of fear or horror	Feelings of fear or horror at the time	2.73	1.70–4.40
Employment	Working full or part-time	Retired/sickness benefits	1.94	1.32–2.84

CIDI, Composite International Diagnostic Interview; TSES-R2, Traumatic Stress Exposure Scale-Revised 2.

a Logistic regression model statistics: Hosmer & Lemeshow *χ* ^2^=4.12, d.f.=6, *P*=0.66. −2LL=777.80. Model correctly classified 82.4% of study participants. Age, marital status, income, age at deployment and number of deployments were all entered into the model as categorical variables (as categorised in Table 1), but were not found to be significant predictors.

## Discussion

This research has significant implications for understanding and responding to the needs of military personnel internationally following peacekeeping deployments. Most importantly, the findings reveal a concerning rate of psychopathology in peacekeeping veterans, with over a quarter (30%) reporting diagnosable psychiatric disorders. In addition, relatively high rates of comorbidity were apparent, with alarmingly high levels of suicidal ideation. It is noteworthy that these elevated rates of psychiatric morbidity were present 12–24 years after the index peacekeeping deployments, suggesting persistent, long-term disability and distress. Notably, the rates of psychiatric disorder, comorbidity and suicidality in peacekeepers were all found to be significantly higher than those found in the civilian comparator sample. The prevalence rates found here are towards the upper end of the range of previously reported studies examining peacekeeper mental health outcomes using self-report data.^15^ In relation to PTSD more specifically, the rates reported here (16.8%) are also well above the pooled prevalence of 5.3% found in Souza *et al*'s meta-analysis.^15^

Importantly, however, the rates of psychiatric disorder reported in this study are comparable with mental health outcomes of combat veterans drawn from methodologically rigorous studies using structured interview methods. Such studies of Vietnam and Gulf War veterans report overall prevalence rates of between 17% and 33%,^1,6,21^ with the extent of psychiatric disorder reported in this study at the upper end of this range. Similarly, the 17% PTSD rate reported here is at least comparable with the 15% and 12% respectively in the USA and Australian Vietnam veteran studies and US personnel who had been deployed to Iraq or Afghanistan (12.9% and 6.2% respectively).^5^ Finally, the 30% rate of psychiatric morbidity reported in this study far exceeds the 14.9%^9^ and 21%^8^ prevalence rates reported across population studies of active military services. These comparisons with both active military and veteran samples are of importance since they suggest that peacekeeping deployments may, in fact, be more pernicious with regard to some mental health outcomes than combat deployments. This is of importance in understanding and delivering support to this unique population.

As might be expected, the peacekeepers were relatively mentally healthy prior to being deployed, with significantly lower incidence of all psychiatric disorders, other than alcohol use disorders, than their civilian counterparts. This is not surprising in the context of rigorous selection processes. These low rates prior to deployment, however, make the poor mental health outcomes for peacekeepers even more striking. The findings also highlight that peacekeepers are exposed to significant levels of lifetime- and deployment-related PTEs. Given that the majority (74%) of this cohort had deployed only once (and therefore their only active deployment had been a peacekeeping deployment), this reinforces the fact that modern-day peacekeeping deployments are frequently hazardous.^10–12^

Indeed, in the peacekeeper cohort, psychiatric disorder was most strongly and consistently associated with high levels of exposure to PTEs and to fear and/or horror related to these PTEs. Number of deployments was a predictor only for major depressive episode, with peacekeepers deployed more than once having twice the risk of current major depressive episode. Deployment type did not appear to be related to mental health outcomes, with personnel deployed to the different peacekeeping missions included in this study showing no significant differences in rates of psychiatric disorder. These findings are consistent with previous findings which suggest that it is not the number or type of deployments *per se* that are the most important factors; rather, it is the actual experiences of PTEs on those deployments that increase risk for subsequent disorder.^8,14^ The finding of the major depressive episode-specific risk of multiple deployments indicates, however, that although not PTE-related, there may be a cumulative emotional impact associated with repeated dislocation from life at home.

### Implications

Given the increasing international operational tempo in peacekeeping operations, the finding that peacekeeping missions are associated with substantial risk for subsequent psychiatric morbidity and distress is concerning and makes it imperative to assertively address this issue in current and future peacekeeping deployments. The needs of peacekeepers should be recognised within initiatives aimed at improving the mental health of military personnel. Initiatives focusing on prevention, early recognition and easy access to evidence-based treatments are a high priority for peacekeeper cohorts. The level of suicidality is of particular concern and signals the importance of specifically targeting existing mental health and suicide prevention programmes to peacekeepers and veteran peacekeepers. It is important that treating clinicians are educated regarding the potential nature and impact of peacekeeping missions so that timely diagnosis and treatment are offered.

The particularly elevated rates of PTSD in peacekeepers relative to active military and veteran comparators suggest that trauma-focused treatments should be central to interventions with this population, whereas more general mental health prevention and intervention strategies may be applicable across all military deployments (including for peacekeepers). The level of suicidality is of particular concern and signals the importance of specifically targeting existing mental health and suicide prevention programmes to peacekeepers and veteran peacekeepers.

A final implication of the current findings is that of service planning for the future. Given the duration of illness, it is unreasonable to expect substantial drops in disorder prevalence over the coming years. Thus, it is reasonable to assume that over a quarter of peacekeeper veterans are likely to continue to have a need for some kind of mental healthcare and support over the long term. As this group continues to age, they will present particular challenges for care. Budgetary and service development projections should bear this in mind.

### Strengths and limitations of the study

The design of this study addresses many of the gaps in the current literature regarding peacekeeper mental health outcomes. The use of a civilian comparator sample provides some context for understanding the levels of psychiatric morbidity in this peacekeeper sample. The study also provides data on the longer-term mental health outcomes of peacekeepers, which have been lacking in the existing evidence base. The adoption of structured clinical interviews is consistent with international best practice for this type of research and provides high-quality data regarding psychiatric diagnoses, overcoming some of the shortfalls of relying on self-report data alone.

Whereas response rates were comparable in comparison with many other veteran health studies, a substantial number of eligible peacekeepers did not participate. There were some significant differences between study participants and non-responders in age, socioeconomic background and deployment, suggesting that study participants may not have been fully representative of the whole peacekeeper population. Most importantly for the current research question, the mental health status of those who chose not to participate is not known; thus, it is possible that veterans with more severe psychiatric morbidity may have chosen not to participate. It is of note that veterans from Somalia and East Timor showed a lower response rate. These deployments have been described to have been particularly challenging for peacekeepers, leaving the possibility that there is a group of highly distressed veterans missing from this analysis.

The cross-sectional nature of the study, although providing a valuable snapshot of the population at this point in time, does not inform our understanding of the course of mental health conditions over the time since deployment and limits conclusions regarding causation. It also means that data on putative risk factors, such as trauma exposure, were obtained retrospectively and may therefore be affected by recall bias. The use of ICD-10 criteria (in preference to DSM), although providing a direct comparison with a matched sample from the Australian community, indicates a need for caution when comparing with studies that have used DSM.

It is important to emphasise that, over the 10–25 years since the deployments reported in this study, mental health policy and practice in the ADF and DVA (and, indeed, in Defence Forces around the world) has changed substantially. Thus, caution is required in generalising these findings to the current generation of military personnel deployed on peacekeeping missions.

In summary, this study builds on the small but growing body of rigorous empirical research examining the psychiatric consequences of peacekeeping operations. The study demonstrates significant elevations for peacekeepers in prevalence of PTSD, generalised anxiety disorder, major depressive episode, alcohol use disorders and suicidality. The findings of this study have considerable implications for service planning and delivery of care to veterans of peacekeeping deployments.
